# A Novel Germline *CDKN1B* Mutation Causing Multiple Endocrine Tumors: Clinical, Genetic and Functional Characterization

**DOI:** 10.1002/humu.21354

**Published:** 2010-11

**Authors:** Sara Molatore, Ilaria Marinoni, Misu Lee, Elke Pulz, Maria Rosaria Ambrosio, Ettore C degli Uberti, Maria Chiara Zatelli, Natalia S Pellegata

**Affiliations:** 1 Institute of Pathology, Helmholtz Zentrum München85764 Neuherberg, Germany; 2 Section of Endocrinology, University of Ferrara44121 Ferrara, Italy

**Keywords:** Multiple endocrine neoplasia type 4, p27, *CDKN1B*, mutation screening, functional characterization

## Abstract

Multiple endocrine neoplasia (MEN) syndromes are characterized by tumors involving two or more endocrine glands. Two MEN syndromes have long been known: MEN1 and MEN2, caused by germline mutations in *MEN1* or *RET*, respectively. Recently, mutations in *CDKN1B*, encoding the cyclin-dependent kinase (Cdk) inhibitor p27, were identified in patients having a MEN1-like phenotype but no *MEN1* gene mutations. Currently, the molecular mechanisms mediating the role of p27 in tumor predisposition are ill defined. We here report a novel germline missense variant in *CDKN1B* (c.678C>T, p.P69L) found in a patient with multiple endocrine tumors. We previously reported a nonsense p27 mutation (c.692G>A, p.W76X) in two patients with MEN1-like phenotype. Functional assays were used to characterize p27P69L and p27W76X *in vitro*. We show that p27P69L is expressed at reduced level and is impaired in both binding to Cdk2 and inhibiting cell growth. p27W76X, which is mislocalized to the cytoplasm, can no longer efficiently bind Cyclins-Cdks, nor inhibit cell growth or induce apoptosis. In the patient's tumor tissues, p27P69L associates with reduced/absent p27 expression and in one tumor with loss-of-heterozygosity. Our results extend previous findings of *CDKN1B* mutations in patients with MEN1-related states and support the hypothesis of a tumor suppressor role for p27 in neuroendocrine cells. © 2010 Wiley-Liss, Inc.

## INTRODUCTION

We recently demonstrated that germline mutation in *Cdkn1b* (MIM# 600778), which encodes the p27 cyclin-dependent kinase (Cdk) inhibitor, causes a multiple endocrine neoplasia (MEN)-like syndrome in the rat (Pellegata et al., 2006). Following this discovery, we identified a germline heterozygous nonsense mutation at codon 76 (c.692G>A, p.W76X) of the human *CDKN1B* gene in several members of a family with a variant of the MEN1 syndrome, thereby demonstrating that *CDKN1B* is a tumor susceptibility gene also in humans (Pellegata et al., 2006). Subsequently, an independent study identified a germline *CDKN1B* frameshift mutation at codon 25 (c.59_77dup, p.K25fs) in a female patient with small-cell neuroendocrine cervical carcinoma, Cushing's disease, and hyperparathyroidism (Georgitsi et al., 2007). This mutation was associated with loss-of-heterozygosity (LOH) of the wild-type *CDKN1B* allele in the patient's cervical carcinoma. Recently, three potentially pathogenic changes in *CDKN1B* (c.-7G>C; c.283C>T, p.P95S; c.595T>C, p.X199QextX*60) were identified in patients with hereditary predisposition to MEN1-like tumors or primary hyperparathyroidism (1°HPT) (Agarwal et al., 2009). *In vitro*, both c.-7G>C and X199Q variants were expressed at reduced level compared to wild-type p27 (p27wt), while p27P95S showed decreased binding affinity for Grb2, an adaptor protein involved in the activation of Ras signalling (Moeller et al., 2003). Therefore, *CDKN1B* mutations, although rare, do occur in patients with MEN1-related states, the so called MEN4 syndrome (MIM# 610755). Due to the small number of mutation-bearing patients so far reported, the phenotypic features associated to MEN4 are still undefined. Moreover, the limited molecular characterization of the naturally-occurring *CDKN1B* mutations identified hinders a thorough understanding of the role of p27 in neuroendocrine tumor predisposition.

The aim of our study was to search for new *CDKN1B* mutations and, if found, to functionally characterize them *in vitro*. We report here a novel *CDKN1B* mutation found in a patient with multiple endocrine tumors. Functional analysis of two mutated p27 proteins (the novel we identified and one we previously reported) regarding subcellular localization, protein binding and degradation kinetic, growth suppression and apoptosis are presented.

## METHODS

### Patients and DNA analysis

The study was approved by the Ethics Committee of the University of Ferrara, Italy and informed written consent was obtained from all patients. Twenty-seven Italian patients displaying a MEN1-like phenotype (hyperparathyroidism, neuroendocrine tumors, pituitary adenoma), but lacking a *MEN1* gene mutation, were screened for mutations in *CDKN1B* (GenBank entry NM_004064.3) as previously reported (Pellegata et al., 2006). To determine the frequency of the c.678C>T allelic variant found in an Italian patient we obtained and analyzed unrelated control DNA samples from the Institute of Human Genetics, Helmholtz Zentrum München, which comprise 370 healthy Germans (KORA Study Group) (Filipiak et al., 2001). Loss of heterozygosity (LOH) analysis was performed as previously reported (Shyla et al., 2010) using the chromosome 12p markers D12S391, D12S358, and D12S1580. Nucleotide numbering reflects cDNA numbering with +1 corresponding to the A of the ATG translation initiation codon in the reference sequence, according to journal guidelines (http://www.hgvs.org/mutnomen). The initiation codon is codon 1.

### Plasmid constructs and antibodies

The two described mutations were introduced by site-directed mutagenesis (Quikchange II Site-Directed Mutagenesis Kit, Stratagene) in the wild-type human *CDKN1B* cDNA cloned in a pEYFP backbone, kindly provided by J. Slingerland (University of Miami, FL). Primary antibodies used were: anti-p27 (clone 57) and anti-GFP tag (BD Biosciences); anti-Skp2 (H-345); anti-Cdk2 (D-12); anti-Cdk4 (C-22); anti-CyclinD1 (H-295); anti-Cyclin E (M-20); anti-α-tubulin (all from Santa Cruz Biotechnology).

### Cell culture, transfections, drug treatments

HeLa and MCF7 cell lines were respectively maintained in DMEM or RPMI 1640 media supplemented with 10% fetal bovine serum, 20 mM L-glutamine, 100 units/ml of penicillin G sodium, and 100 µg/ml streptomycin (all Invitrogen). The GH3 cell line was maintained in F-12K medium supplemented with 15% horse serum, 2.5% fetal bovine serum, 20 mM L-glutamine, 100 units/ml of penicillin G sodium, and 100 µg/ml streptomycin.

For p27 half-life determination in exponentially growing cells, cycloheximide (CHX, Sigma- Aldrich, 25 µg/ml) was added to the cells for the indicated times.

Transfections were carried out as already reported (Pellegata et al., 2006). When establishing stable transfectants, GH3 cells were selected by adding 0.4 mg/ml G418 to the culture medium for 2-3 weeks and individual colonies picked and expanded in presence of G418.

### RNA interference

For RNAi studies, short interfering RNA (siRNA) duplexes specific for *SKP2* (n=4; siGenome SMARTpool, Dharmacon) and *KPC1* (n=2; ID 133486, 133487 Ambion) and scrambled siRNA (Ambion) were obtained and transfected into MCF7 cells using X-tremeGENE reagent (Roche) together with 0.03 µg of plasmid DNA.

### Protein extraction, immunoblotting and immunoprecipitations

Protein extraction and immunoblotting were carried out as already reported (Pellegata et al. 2006).

For IP, 500 µg of total protein were incubated with 5 µl of anti-YFP tag antibody (Clontech-Takara Bio Europe) and 20 µl of agarose resin (Santa Cruz) overnight at 4°C. For GST-pull down assay, 500 µg of total protein were incubated overnight at 4°C with 2 µg of recombinant Cdk2-GST (Abnova) and 20 µl of Glutathione Agarose (Santa Cruz).

### Intracellular localization and apoptosis assay

p27 knock-out (-/-) MEFs were obtained from 13 days old embryos upon trypsin digestion for 15 min at 37°C. Cells were grown in DMEM supplemented with 10% fetal bovine serum, 20 mM L-glutamine, 100 units/ml of penicillin G sodium, and 100 µg/ml streptomycin (all Invitrogen). For localization experiments, MEFs were grown on coverslips, transfected and processed for immunocytochemistry as described (Pellegata et al., 2006) with minor modifications. For the apoptosis assay, HeLa cells were transfected while exponentially growing on coverslips. At 24h or 48h post-transfection cells were fixed and nuclei were stained with DAPI.

### Clonogenic assay and growth curve

To examine clonogenic activity, GH3 cells were plated (500,000 per well) in 6-well plates and transfected with p27wt, p27W76X or p27P69L using Fugene-6 (Roche). The day after the transfection, the cells were diluted 1:6 and, starting from the day after, selected for 6 weeks by adding 0.4 mg/ml G418 to the culture medium and stained with 0.3% crystal violet in 30% ethanol. Colonies that contained ≥50 cells were scored.

p27W76X GH3 stable clones, and untrasfected GH3 cells were plated in duplicate (100,000 per well) in 12-well plate, in full medium plus 400 µg/ml G418. The number of cell was counted every two days over a 9 day-period using a cell counter.

### Immunohistochemistry

The two described mutations were introduced by site-directed mutagenesis in the wild-type human *CDKN1B* cDNA cloned into the pEYFP (YFP, yellow fluorescent protein) backbone. Immunohistochemistry were performed using a monoclonal anti-p27 antibody (BD Biosciences) as already reported (Pellegata et al., 2006).

## RESULTS

### Germline genetic changes and clinical presentation

**Figure 1:**
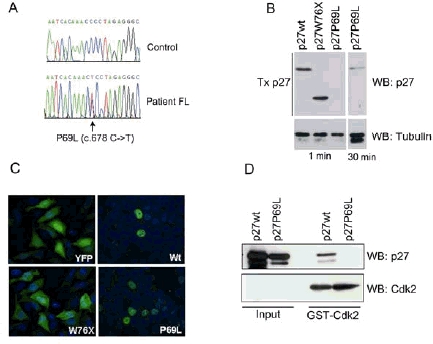
Characterization of the P69L and W76X mutations in p27. A, Sequence chromatograms showing the novel nucleotide change identified in patient FL compared to a control normal individual. B, Expression of the p27W76X and p27P69L mutant proteins 24h after transfection of MCF7 cells. Western blotting (WB) was performed with a monoclonal anti-p27 antibody. C, Localization of the p27 proteins. Asynchronously growing p27-negative MEFs were transiently transfected with the different YFP fusion constructs. Cells were fixed 24h later and nuclei were counterstained with DAPI. Cells were examined for direct YFP fluorescence. D, p27P69L does not associate with Cdk2. Lysates obtained upon transfection of HeLa with p27wt or p27P69L were incubated with purified GST-Cdk2 protein. Following GST pull-down, 50 µgr of the original lysate (Input) and the entire pull-down (GST-Cdk2) were analyzed by Western blotting (WB) with antibodies against p27 or Cdk2.

We screened the genomic DNA of 27 patients for *CDKN1B* germline changes and found a previously unreported variant at codon 69 (c.678C>T, p.P69L) in patient FL, a 79-year-old Caucasian female (Fig. 1A). This change was not reported in the single nucleotide polymorphism (SNP) database and was not observed in 370 unrelated healthy Caucasian controls, confirming that it is neither a polymorphism nor a rare variant. This patient presented with bilateral multiple lung metastases of bronchial carcinoid and type 2 diabetes mellitus. A sellar magnetic resonance imaging (MRI) revealed a pituitary microadenoma. Clinical work-up did not display any alteration in pituitary hormone levels, indicating the presence of a non functioning pituitary microadenoma. This patient had a history of endocrine and non-endocrine malignancies: at age 67 she was admitted for surgical resection of multiple typical bronchial carcinoids and a subcutaneous epigastric lipoma, and she was subjected to left parathyroidectomy because of a parathyroid adenoma causing 1°HPT. At age 64 a total thyroidectomy had been performed due to a papillary thyroid carcinoma with neck lymphnode metastases (pT1bN1M0). The patient had no family history of endocrine tumors. Her relatives refused to undergo genetic testing and their phenotype could not be determined.

### Functional characterization

In our previous work we identified a heterozygous nonsense mutation at codon 76 (W76X) in a proband with GH-secreting pituitary adenoma and 1°HPT, and in her sister displaying renal angiomyolipoma (Pellegata et al., 2006). Upon transfection into normal rat fibroblasts, a Myc-tagged p27W76X protein localized to the cytoplasm (Pellegata et al., 2006). Although the renal tumor showed no p27 expression we have now detected p27W76X expression in the normal kidney of the mutation-positive woman, where it localizes to the cytoplasm.

To investigate which functional properties of p27wt are affected by P69L and W76X changes, we generated plasmid vectors that expressed both p27 mutants as fusion proteins with the YFP tag located at the N-terminus (Connor et al., 2003). Transfection of these constructs in MCF7, HeLa and the p27-negative GH3 pituitary adenoma cells confirmed the expression of the fusion proteins *in vitro* and showed that p27P69L is consistently expressed at reduced level compared to p27wt in all cell lines (Fig. 1B).

Following transfection of exponentially growing p27-/- mouse embryonal fibroblasts (to avoid any possible interaction with endogenous p27), p27P69L localized mainly to the nucleus, like p27wt (Fig. 1C), although there was a higher proportion of cytoplasmic localization (average of YFP-positive cells showing cytoplasmic localization of the transfected p27P69L protein: 22.6%) compared to p27wt (average of YFP-positive cells showing cytoplasmic localization of the transfected p27wt protein: 8.2%). As expected, p27W76X was cytoplasmic (Fig. 1C).

**Figure 2:**
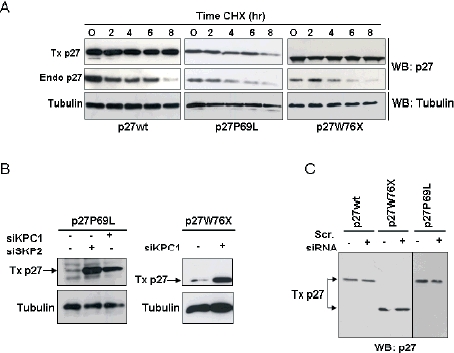
Degradation kinetics of the p27W76X and p27P69L mutant proteins and pathways of degradation. A, p27P69L is slightly more unstable than p27wt. Asynchronously growing MCF7 cells were transiently transfected with the different constructs and 24h later treated with CHX for the indicated times. Western blotting (WB) was performed using an anti-p27 antibody. The transfected (Tx) exogenous p27 proteins and the endogenous (Endo) p27 are indicated on the left. To control for equal loading the membranes were probed with anti-α-tubulin antibody. B, Degradation of p27W76X and p27P69L is in part mediated by Skp2 and KPC1. MCF7 cells were transfected with siRNA against Skp2 or KPC1 together with expression plasmids for YFP-p27wt, -p27W76X and -p27P69L and analyzed 48h later by western blotting for p27. Transfected (Tx) exogenous p27 proteins and the endogenous (Endo) p27 are indicated on the left. C, Scrambled siRNA (Scr. siRNA) has no effect on the expression of p27 proteins. Only the transfected proteins are shown.

p27P69L is consistently expressed at lower steady-state level compared to p27wt. This could be due to enhanced protein degradation, the mechanism responsible for p27 down-regulation in many human tumors (Pagano et al., 1995; Loda et al., 1997). Exponentially growing MCF7 cells transfected with p27wt, p27P69L or p27W76X were incubated with Cycloheximide (CHX) to block new protein synthesis and analyzed at different time points thereafter. Up to 8h post-treatment we observed no change in p27wt and p27W76X amount, while p27P69L showed reduced expression after 6h (Fig. 2A). This indicates that p27P69L is degraded slightly faster than p27wt.

siRNA-mediated knock-down of the ubiquitin ligases that mediate p27wt degradation (i.e. SKP2, which acts in the nucleus in G1-S, and KPC1, which works in early G1 in the cytoplasm) (Carrano et al., 1999; Kamura et al., 2004) showed that either both these degradation pathways, or just *KPC1*, play a role also in the degradation of p27P69L and p27W76X, respectively (since p27W76X is cytoplasmic, we only tested the effect of *KPC1* knockdown) (Fig. 2B). Co-transfection of the p27 proteins with scrambled siRNA confirmed the specificity of the *siSKP2* and *siKPC1* molecules (Fig. 2C).

Considering the crystal structure of the p27-CyclinA-Cdk2 protein complex, the P69L change affects one of the six amino acids involved in the direct binding to Cdk2 (Russo et al., 1996). To verify whether this mutation indeed alters the binding of p27 to Cdk2, we performed Cdk2 pull-down assays using HeLa cells transiently-transfected with p27P69L or control p27wt. Associated p27 proteins were detected by immunoblotting. While p27wt binds Cdk2, p27P69L does not bind to the kinase (Fig. 1D).

**Figure 3:**
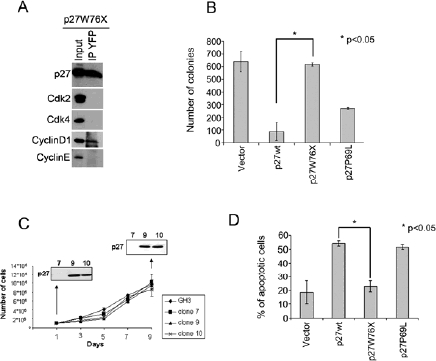
Effect of the p27 mutations on protein binding, cell growth and apoptosis. A, Asynchronously growing HeLa cells were transfected with p27W76X and collected 24h later. Five hundred microgr of the lysate were immunoprecipitated (IP) o.n. with the anti-YFP antibody. Fifty microgr of the original lysate (Input) and the entire IP (IP YFP) were analyzed by Western blotting (WB) with antibodies against the indicated proteins. B, p27-negative GH3 cells were transfected with the indicated expression plasmids and then selected in G418-containing media for 3 weeks. Colonies were stained and counted. The values represent the mean of 2 independent experiments (each consisting of 6 separate plates) ±SD. C, Growth curves of representative GH3-derived clones expressing p27W76X. At day 1 and day 9 cells were lysed and total proteins were analyzed by Western blotting (WB). Values are the mean of 2 independent experiments ±SD. D, p27wt, p27W76X and p27P69L fusion proteins were transiently transfected into HeLa cells and 48h later stained with DAPI. Cells positive for YFP (=transfected) and showing chromatin condensation (=apoptotic) were counted and expressed as the percentage of the YFP-positive cells without features of apoptosis (Y axis). The values represent the average of 6 independently-transfected plates ±SD.

p27W76X retains most of the cyclin/Cdk binding domain of p27 (aminoacids 28 to 90), therefore it could bind to and sequester in the cytoplasm the canonical p27 partners: Cyclins E and D1, Cdk2 and Cdk4. Upon transfection of proliferating HeLa cells, we immunoprecipitated (IP) p27W76X and detected interacting Cyclins and Cdks by immunoblotting with specific antibodies. A minimal fraction of p27W76X binds to Cyclin D1, while there is no association with the other molecules (Fig. 3A). Similar results were also obtained using GH3-derived clones stably and constitutively expressing p27W76X (see below), demonstrating that the lack of association with partner proteins is not due to transient p27W76X expression nor to interference by endogenous p27wt.

p27 is a negative regulator of cell cycle progression, and its loss is associated with disease progression and unfavourable outcome in many cancer types (Chu et al., 2008). To determine the effect of P69L and W76X mutations on cell growth, we performed a clonogenic assay using p27-negative GH3 cells. The results showed that p27wt inhibits the growth of these cells while p27W76X does not. p27P69L was less efficient than p27wt at suppressing cell growth (Fig. 3B).

To verify that the long-term expression of p27W76X has no effect on cell proliferation, we picked and expanded several stably-transfected, GH3-derived clones. Selected representative clones were employed for growth curves over a period of 9 days. In agreement with the clonogenic assay results, constitutive overexpression of p27W76X did not affect GH3 cell proliferation (Fig. 3C).

Tumor suppression by p27wt works through inhibition of cell proliferation, but also induction of apoptosis in some cell types (Katayose et al., 1997). We assessed whether W76X and P69L mutations interfere with the pro-apoptotic activity of p27wt using HeLa cells as experimental model, as these cells undergo apoptosis upon p27wt overexpression (Nickeleit et al., 2008). DAPI staining showed that p27P69L expression induced apoptosis at both 24h and 48h post-transfection, similarly to p27wt, whereas p27W76X lost this ability (Fig. 3D).

### p27 expression in patients tissues

**Figure 4:**
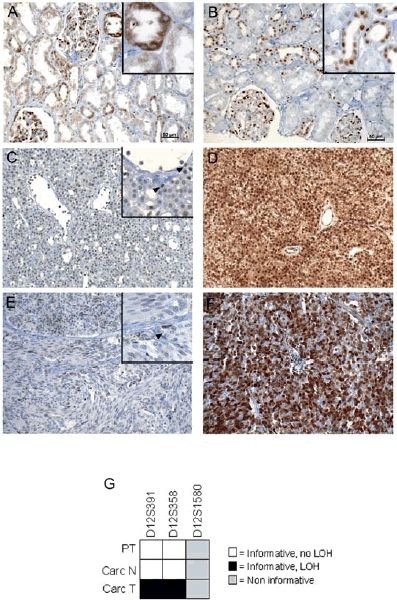
Expression of p27P69L and p27W76X in patient tissues. A-F, Immunohistochemical staining with an anti-p27 specific antibody. A-B, Staining of the normal kidney tissue from the W76X mutation-positive patient who developed renal angiomyolipoma (A), and from a normal control individual (B). The patient with the germline p27 mutation shows cytoplasmic staining in the renal tubules, whereas the normal individual only shows nuclear p27 immunoreactivity in some tubules. C-D, Staining of the parathyroid tumor from the P69L mutation-positive patient (C) and of a similar tumor from a sporadic mutation-negative patient (D). The p27 immunoreactivity of the tumor cells in the mutation-positive patient is dramatically reduced compared to the interspersed endothelial cells (indicated by arrows in the Inset). E-F, Staining of one bronchial carcinoid from the P69L mutation-positive patient (E) and of a similar tumor from a sporadic patient without mutation in p27 (F). There is virtually lack of p27 immunoreactivity of the tumor cells of the mutation-positive patient compared to the interspersed endothelial cells (indicated by an arrow in the Inset). Bar, 50 µM. Original magnification: A-F, 200X; Insets, 400X. G, LOH analysis of the tissues of the P69L mutation-positive patient for 3 micro satellite markers adjacent to the p27 locus. PT, parathyroid tumor; Carc N, normal tissue adjacent to the bronchial carcinoid; Carc T, bronchial carcinoid (tumor tissue).

We analyzed the expression of p27 in the parathyroid adenoma and one bronchial carcinoid of the patient carrying the p27P69L mutation using IHC. Parathyroid adenoma cells showed weak p27 nuclear staining in <1% of the tumor cells (interspersed normal endothelial cells served as controls) (Fig. 4C-D). The parathyroid adenoma of a mutation-negative control individual exhibited strong p27 nuclear positivity in virtually all cells. A bronchial carcinoid of the P69L mutation-positive patient showed virtually no p27 staining when compared to a bronchial carcinoid of a mutation-negative individual (Fig. 4E-F). To determine the cause of the reduced p27 expression level in the tumors of this patient, we extracted DNA from tumor and adjacent normal cells and performed LOH analysis by amplifying three microsatellite markers flanking the p27 locus (at position 12,76 Mb on chromosome 12p): D12S391 (12,34 Mb), D12S358 (12,53 Mb) and D12S1580 (13,23 Mb). We observed LOH for the 2 informative microsatellite markers near the p27 locus in the carcinoid, but not in the parathyroid adenoma (Fig. 4G).

## DISCUSSION

Our current study confirms and extends previous findings concerning the presence of *CDKN1B* germline mutations in a sub-set of patients with multiple endocrine tumors. We identified a novel germline missense mutation in a patient with multiple endocrine and non-endocrine malignancies. Based on few recent studies the incidence of *CDKN1B* mutations in patients with a MEN 1-related phenotype is estimated to be 1.5%-2.8% (Georgitsi et al., 2007; Agarwal et al., 2009). In our patient cohort the incidence was slightly higher (1/27; 3.7%). The identification of additional mutation-positive cases will allow to more precisely assess the contribution of *CDKN1B* mutations to the MEN4 syndrome.

Functional *in vitro* characterization of the newly identified mutation (P69L), as well as of the W76X change, identified an altered molecular phenotype for both encoded proteins.

The P69L mutation affects an amino acid located in the Cdk2 binding domain of the protein (aminoacids 52-93) and this change is predicted with a high degree of confidence to reduce by more than 55% the hydrophobicity of the region (http://www.expasy.org/spdbv/), thereby dramatically altering the environmental conditions necessary for binding to the kinase. Our studies demonstrated that indeed this variant does not bind to Cdk2. The CyclinE/Cdk2 complex is a very important target of p27 and this binding is crucial for p27-mediated regulation of G1 to S phase cell cycle progression (Sherr and Roberts, 1999). Indeed, the inability of p27P69L to bind to Cdk2 associates with impaired growth suppression of GH3 neuroendocrine tumor cells *in vitro*.

We observed extremely reduced/absent p27 expression in the patient's tumors (parathyroid, bronchial carcinoid). This goes beyond the low expression level of p27P69L *in vitro* and suggests that both the wt and mutant allele are expressed at reduced level. Thus, p27P69L retains characteristics which are incompatible with sustained cell proliferation. In the carcinoid, lack of p27 expression was associated to LOH. LOH had also been observed in the tumor of the patient carrying the p27K25fs mutation (Georgitsi et al., 2007). The observation that in several non-endocrine human tumors somatic biallelic inactivation of *CDKN1B* is exceedingly rare led to the hypothesis that p27 is a dose-dependent (haploinsufficient) tumor suppressor (Philipp-Staheli et al., 2001), as formally demonstrated in mice (Fero et al., 1998). In contrast, p27 might function as a canonical tumor suppressor in neuroendocrine cells. Further studies on mutation-positive patients will help clarify this issue.

The patient carrying the P69L mutation shows a relatively late age at onset of endocrine disease. The few patients so far reported who carry germline *CDKN1B* mutations show variable age at onset. The two probands carrying the W76X or the c.59_77dup, p.K25fs mutation developed tumors at relatively young age (36 and 45 years of age, respectively), while all other carriers showed a clinical phenotype at age 50 or older. We speculate that the age at onset of the mutation-carriers might be related to how severely the *CDKN1B* mutation alters the p27 sequence and likely its function, which would in turn provide stronger selective advantage and lead to cell expansion.

The W76X mutation prevents the encoded p27 protein from entering the nucleus, the cell compartment where p27 exerts its function as a cell cycle inhibitor, and indeed p27W76X can no longer inhibit the growth of GH3 cells both by clonogenic assay and by stable expression. Moreover, p27W76X has lost the pro-apototic function of p27wt. This is in agreement with reports showing that cells with cytoplasmic p27 are resistant to apoptosis, but can become sensitive upon relocalization of the protein to the nucleus (Short et al., 2008). Interestingly, the truncated p27W76X protein is stable and can be detected *in vitro* upon transfection but also, and more significantly, in the patient's normal kidney tissue by immunohistochemistry. Although mutated alleles encoding for truncated peptides tend to be unstable and often the encoded peptides are not detected, it has been demonstrated that p27 deletion mutants comprising only the N-terminus of the protein can be expressed *in vitro* (Müller et al., 2000; Fuster et al.,2010).

Although the W76X mutation alters several p27 functions, the only tumor tissue of a mutation-positive patient available for analysis showed no p27 immunoreactivity (Pellegata et al., 2006) while the truncated protein was present in the patient's normal tissue. This indicates that this mutation possesses functions that are detrimental to, or no longer needed by, neuroendocrine tumor cells and therefore selected against during tumor progression. These functions might affect still unidentified mechanisms of p27 action and deserve further evaluation.

Studies on naturally-occurring *CDKN1B* mutations will help us understand the link between p27 and neuroendocrine tumor predisposition, a prerequisite to offer in the future personalized care to the families carrying such mutations.
